# In-Home Older Adults’ Activity Pattern Monitoring Using Depth Sensors: A Review

**DOI:** 10.3390/s22239067

**Published:** 2022-11-23

**Authors:** Md Sarfaraz Momin, Abu Sufian, Debaditya Barman, Paramartha Dutta, Mianxiong Dong, Marco Leo

**Affiliations:** 1 Department of Computer Science, Kaliachak College, University of Gour Banga, Malda 732101, India; 2 Department of Computer & System Sciences, Visva-Bharati University, Bolpur 731235, India; 3 Department of Computer Science, University of Gour Banga, Malda 732101, India; 4 Department of Science and Informatics, Muroran Institute of Technology, Muroran 050-8585, Hokkaido, Japan; 5 National Research Council of Italy, Institute of Applied Sciences and Intelligent Systems, 73100 Lecce, Italy

**Keywords:** classification of sensor data, computer vision, depth imagery, fall detection, gait analysis, HAR, smart home, survey

## Abstract

The global population is aging due to many factors, including longer life expectancy through better healthcare, changing diet, physical activity, etc. We are also witnessing various frequent epidemics as well as pandemics. The existing healthcare system has failed to deliver the care and support needed to our older adults (seniors) during these frequent outbreaks. Sophisticated sensor-based in-home care systems may offer an effective solution to this global crisis. The monitoring system is the key component of any in-home care system. The evidence indicates that they are more useful when implemented in a non-intrusive manner through different visual and audio sensors. Artificial Intelligence (AI) and Computer Vision (CV) techniques may be ideal for this purpose. Since the RGB imagery-based CV technique may compromise privacy, people often hesitate to utilize in-home care systems which use this technology. Depth, thermal, and audio-based CV techniques could be meaningful substitutes here. Due to the need to monitor larger areas, this review article presents a systematic discussion on the state-of-the-art using depth sensors as primary data-capturing techniques. We mainly focused on fall detection and other health-related physical patterns. As gait parameters may help to detect these activities, we also considered depth sensor-based gait parameters separately. The article provides discussions on the topic in relation to the terminology, reviews, a survey of popular datasets, and future scopes.

## 1. Introduction

The number of older adults (seniors) is increasing globally and different epidemics and pandemics are frequently arising that place pressure on global healthcare infrastructure [1,2,3]. These challenges largely affect seniors. Since seniors who live alone often encounter problems such as falls, breathing issues, heart attacks, etc., they have been greatly affected by inadequate health care facilities. Among these, falls are a common problem for seniors, which may cause serious health issues [4,5,6]. As the required care is dependent on the response and rescue time, falls need to be detected as quickly as possible to prevent any substantial damages to health. Due to the steady increase in the number of seniors, existing health care infrastructure as well as the number of trained medical professionals have been proven to be insufficient [7,8].

Therefore, in-home care systems for seniors are an attractive solution. These systems are also effective for patients who are in the recovery phase. Monitoring is the primary part of care; however, engaging a human to monitor the activity pattern of a senior 24 × 7 is a tedious task. Fortunately, it can be achieved using an intelligent system by analyzing the collected data of different sensors in real-time [9,10,11,12,13]. This system can be developed by integrating both wearable and non-wearable sensors. Many systems have been proposed using wearable sensors [14]. However, wearing sensors 24 × 7 is very uncomfortable and sometimes the person may forget to wear these. Additionally, wearable sensors need to be carefully used to prevent any damage. The CV technique with sensors and IoT devices can leverage state-of-the-art activity detection algorithms to complete this task in non-intrusive or un-obtrusive ways [15,16]. However, these visual sensors, especially RGB cameras, may compromise privacy; the size of the RGB frame is also high. To mitigate this problem, depth, thermal, and audio sensor imagery could be used [17,18,19]. Due to the larger scope of the area, this article focuses on only depth sensor-based approaches. The depth sensor measures the distance of the object and shows the 3D shape of the object with different color intensities at different distances [20]. After the real-time depth video data are captured, they need to be analyzed. To process and analyze those data, cloud computing is generally used [21,22]. However, a significant amount of time is required to respond, and high bandwidth data communication is necessary. Moreover, there exist data security and privacy issues. These challenges could be addressed if the data are processed close to the origin. It may minimize the response time as well as the network overload. Thus, a monitoring system can incorporate edge computing to process data near the source [16,23]. These edge devices later interact with the cloud to complete the whole process. Several articles show that the analysis of some gait parameters could provide a fall risk assessment as well as an assessment of other activity [6]. In this paper, we review fall detection and other health-related activity pattern analyses by further classifying them, according to the use of gait features. We also study machine learning, deep learning and other feature-engineering methods to see which of these approaches has been used most frequently.

### Contributions of This Article

In this article, we focused on the computing perspective of the problem and reported several state-of-the-art techniques which use depth sensors-based data. Depending on the broad objectives, these techniques may be grouped into the following two categories: human fall detection and activity pattern analysis. These technique mostly use either the depth image classification technique without gait parameter or with gait parameter. The following are the contributions of our article:

- A discussion on why in-home care monitoring systems using depth sensors are relevant;
- A systematic review on state-of-the-art computing techniques for in-home monitoring systems for seniors based on depth data;
- Survey on benchmark depth information datasets related to in-home seniors’ activities;
- Discussion on future directions and potential ideas for future research.

The rest of the paper is organized as follows. We first introduce the terminology and background in Section 2, followed by a review on the state-of-the-art, Section 3 presents a detailed review of fall detection and an activity analysis which is further classified into two subsections. Related benchmark datasets are reported in Section 4. Discussions on state-of-the-art techniques and possible future scopes are included in Section 5. Finally, Section 6 presents the conclusion.

## 2. Terminology and Backgrounds

This section introduces relevant terminology and the background of our focused study.

### 2.1. In-Home Monitoring Systems for Seniors

Monitoring is the most essential part of any in-home care system for seniors. The approach can be either intrusive or non-intrusive. Different types of wearable sensors are generally used in the intrusive approach, whereas CV-based techniques are deployed in the non-intrusive approach. In CV-based techniques, depth or thermal imagery is preferable over RGB due to privacy issues. Additionally, in-house local computation may also introduce privacy as well as latency issues.

**Figure 1:**
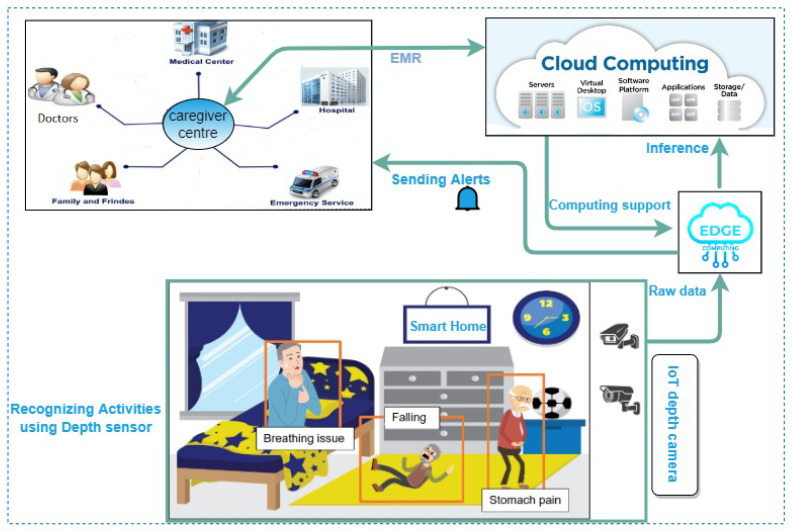
A working overview of a smart home for senior care.

A typical working pipeline is shown in Figure 1, where depth sensor-based cameras are used to monitor seniors 24 × 7 with the help of edge-IoTs.

This is a scenario of a smart home with an older adult; a depth sensor camera has also been connected for monitoring, which is basically an edge device. It collects raw data, processes them in a local computer and if any dangers are detected, it sends alerts to the nearest caregiver centre or hospitals as well as the concerned relatives. It also sends results to the cloud for future uses. This approach might be helpful to resolve the latency issue. Despite privacy concerns, depth sensor cameras have been used which can recognize activities such as falls, abnormal breathing, chest or head pain, and so on. Here, our main focus is on fall detection as well as other activity pattern analyses using depth imagery and gait analysis. We discuss the detection of falls along with several other damaging events in the next section.

#### 2.1.1. Human Fall

Due to the rise in the number of seniors in developing countries [24,25], in-home-care and patient monitoring systems have gained increased attention. Although researchers are constantly trying to improve the system, we still have a long way to go [26,27,28]. Sometimes, we fall unintentionally due to abnormal health conditions. This may become fatal for seniors. It may lead to serious health problems or even death. So, the fall detection mechanism is an essential feature for any in-home-care patient monitoring system. Therefore, we focus on fall detection using depth sensors including a gait analysis [17,29,30,31].

#### 2.1.2. Other Elderly Activities

Besides falls, other physical health-related events, such as daily patterns, mobility, heart attack, breathing patterns, etc., need to be analyzed too. The modern AI-based computer vision techniques can predict damaging events for an elderly or a patient by analyzing their activity pattern [32,33,34]. Since seniors often need 24 × 7 care facilities, a continuous activity analysis could detect probable health problems such as heart attacks, pain, etc. In activity analysis, daily activities (e.g., standing, sitting, walking, sleeping, coughing, eating, etc.), behavior, posture, and facial expressions are recorded and analyzed. When any deviation from the normal pattern has been observed, the system first tries to predict the cause. If it indicates an emergency situation, then an alert is sent to the nearest caregiver centers and hospitals immediately. Emergency contacts, as well as family members, are notified.

### 2.2. Computing

In this smart in-home-health monitoring or care system, various types of computing techniques are used, such as classical machine learning (ML) [35], deep learning (DL) [36,37], edge computing(EC) [38], etc. These computational techniques obtain real-time spatial data (video frames) captured by an installed depth camera inside the home. This paper briefly discusses suitable computing techniques in the following subsections.

#### 2.2.1. Machine Learning

Nowadays, when discussing artificial intelligence (AI), machine learning (ML) algorithms come to mind first; ML algorithms are successfully applied in various domains. It is a subfield of AI which largely depends on data and their features. The term ML itself explains that machines can learn from data and features [35,39]. It helps the system to learn and improve from past observation without being explicitly programmed. Generally, classical ML techniques use hand-crafted features; therefore, the method is sometimes referred to as feature-based learning. There exist many ML algorithms in the literature and the algorithmic techniques have been improving day by day. Some classical algorithms are: Linear Regression [40], Decision Tree [41], Support Vector Machine (SVM) [42], etc. There are many applications that use ML algorithms, such as speech recognition, traffic prediction, product recommendation, healthcare delivery, etc. [43,44,45].

#### 2.2.2. Deep Learning

Deep Learning (DL) is a data-driven ML technique [36,37]. The term deep usually refers to the number of hidden layers in the network. The deeper we go, the more features we obtain, and the greater the accuracy that is achieved. Therefore, more data are required for training. In order to handle more data, more computational power is required. Nowadays, technologies that can facilitate the level of power required, such as GPU(graphics processing unit), make DL highly popular. The advantage of using DL over the classical ML algorithm is that it solves many complex problems with better accuracy and requires less human intervention as it extracts features automatically.

The most popular DL algorithms are: Convolutional Neural Network (CNN) [46] for CV, Recurrent Neural Network (RNN) and Long Short-Term Memory (LSTM) Networks for Natural Language Processing (NLP), etc., [47,48]. Most of the advanced applications are: self driving cars, smart healthcare, virtual assistants, etc., [49,50]. Additionally, many application areas will be enhanced in the near future based on this DL-based computational technique due to its higher accuracy [43,51].

#### 2.2.3. Edge Computing

Internet of things (IoT) [52] systems include multiple interconnected devices with various sensing capabilities. These devices have become a part of our daily life and generate a huge amount of data. Cloud computing [53,54] is used to process this huge amount of data. However, cloud computing introduces an unwanted delay in the computing process. Moreover, it has some privacy as well as security issues and has higher costs too.

Edge computing (EC) and fog computing [55,56] have often been used to overcome these issues. Both Fog and Edge computing share almost the same concept, i.e., to move computing and storage away from the centralized data center (cloud). Edge computing is a distributed computing paradigm focused on bringing computation to the edge (end points), i.e., close to the source of data. Apart from reducing latency, this approach also minimizes bandwidth as well as reducing the overhead of the cloud, and offers better privacy.

Fog computing is an extension of cloud computing and acts as a mediator between the edge and the cloud. When edge devices send huge amounts of data to the cloud server, fog nodes receive the data and analyze them before passing them towards the cloud. Then, the fog nodes transfer the important data and drop the unimportant data or keep them for further processing.

### 2.3. Depth Sensor and Imagery

**Figure 2:**
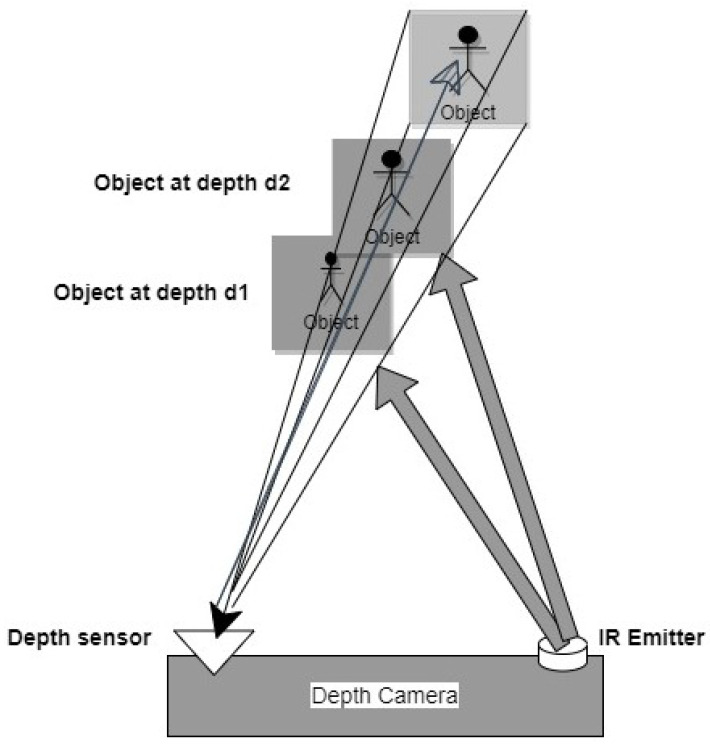
A working scenario of Depth sensor.

As far as privacy is concerned, a depth sensor is a good option over RGB. Additionally, the depth sensor does not require any ambient light. Traditional cameras project the 3D (three-dimensional) world into 2D but depth sensors sense 3D information by measuring the distance from different viewpoints. Depth sensor cameras are basically two cameras in one body. One is a traditional RGB camera, while the other is an infrared (IR) camera. The IR camera is used to measure the distance between the camera and objects in the scene. This information is then used to calculate the depth of field for each image. It acquires multi-point distance information across a wide field of view and provides z-level information of an image. It calculates depth according to the reflection of the light at different points [20]. Depth sensors and their imagery techniques have been applied in several areas where privacy is a concern, such as healthcare [57], facial recognition [58], surveillance [59], etc., [60]. The recent advancement of in-depth sensors in association with CV algorithms makes it more popular among researchers and developers. Microsoft Kinect is an example of a popular depth sensor that is widely used for many purposes [61,62]. Figure 2 shows the formation of a 3D image using a depth sensor by acquiring z information from the image.

It provides the depth intensity value, i.e., the RGB-D value that represents different distances with different colors. It also measures the distance of each point of the object’s body by transmitting invisible near-infrared light and measuring its “time of flight” after it reflects off the objects. It also shows the foreground and background differences of the object. Although, there are some limitations of depth sensors, such as low resolution, short sensing distance, and sensitivity to optical interference [63].

### 2.4. Gait Analysis

Gait analysis is a study of human motion including the comparative motion of different body parts and joints. The gait analysis is also highly effective for in-home senior care, as it produces a clear mobility pattern of seniors [64,65]. The gait analysis could easily be performed using state-of-the-art techniques [66] with depth information. If a patient faces any kind of health issues such as pain, breathing problems, etc., then their gait changes accordingly. Therefore, gait parameters (e.g., speed, cadence, stride length, swing time, weight distribution, etc.) can be used to reduce the risk of falls. Moreover, these parameters can also be used to determine sudden changes while engaged in other activities (e.g., walking, sitting, standing, etc.). Different gait parameters indicate distinct health issues, i.e., gait-speed indicates weakness, postures indicate spinal cord issues or issues with other body parts, etc. A gait analysis might also indicate several other diseases (e.g., progressive dementia, residual hemiplegia, Parkinson’s disease, etc.). It can also recognize symptoms of falling by observing any abnormalities while walking or moving. It also helps to identify any change in posture during movement. It does this by extracting the 3D kinetic joint motion data of humans. A daily gait analysis may help seniors to live independently in their homes. Thus, the early determination of gait might help to improve the quality of life of seniors. It will also be helpful to recognize early symptoms of an oncoming health issue.

**Figure 3:**
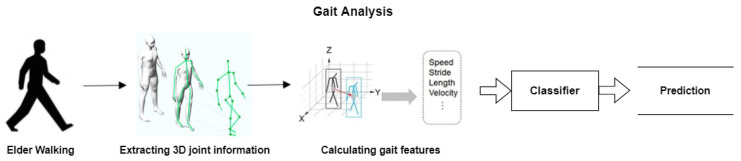
Gait analysis scenario.

Figure 3 shows how a typical gait analysis works by extracting 3D joint information and calculating the gait features of a person. Since gait analysis plays a vital role in any in-home seniors’ care system, a detailed discussion is included in Section 3, along with several potential applications.

## 3. Survey on State-of-the-Art

The objective of this review is to present a systematic study on existing works of in-home seniors’ care using depth sensors, for which the most popular application in this research domain is human-to-fall detection. Moreover, there also exist several works on the detection of other health-related physical activities. So, we defined a methodology as discussed below to present the topics in a systematic way.

In this paper, we have reviewed older adult in-home monitoring approaches based on depth sensors published since 2011. We selected different related papers from the Google Scholar, Web of Science, Scopus, and PubMed databases using different combinations of search keywords. We divided the keywords into two parts. In the first part, we used the keywords related to falls (e.g., ”fall detection using depth sensor”, and “fall detection using depth images”); then, we added the keyword ‘gait’ to these (e.g., “gait-based fall detection using depth images”). In the second part, we used activity-related keywords (e.g., “activity analysis using depth map). Then, we added added the same keyword ‘gait’ to these terms (e.g., “gait-based activity analysis using depth videos”) and performed the search again.

By using all these keywords, we obtained a large number of articles from all four databases. We prioritized those articles that are available in more than one database. In this way, we acquired around two hundred papers. Then, we read them, and manually selected and reviewed the 59 works which fall into the scope of our work. Among these 59 articles, 18 are present in Web of Science, 31 are present in Scopus, 9 are present in PubMed, and all 59 articles are present in Google Scholar. There were 91 more articles available from the above databases, and they were used to identify the problem, highlight related open issues and to validate the information, terms, data, etc. Two separate sections are provided which present existing works on the applications of fall detection and other activity pattern analysis.

### 3.1. Fall Detection

The field of automatic human fall detection has been extensively studied by different researchers over the last two decades [31,67]. A steady increment in computational power led to the development of sophisticated fall detection techniques with a high accuracy. The following two sections discuss several state-of-the-art techniques for fall detection using depth image classification without and with the gait parameter, respectively.

#### 3.1.1. Fall Detection without Gait Parameter

Depth images are frequently used to detect falls. Several depth image classification-based techniques without the gait parameter are reported here.

DL-based Methods: In a study in [68], a video-based fall detection system was proposed by Chen et al. They used a fully convolutional architecture with residual connections that takes a sequence of 3D poses as the input. Their model was trained and evaluated on an NTU RGB+D Action Recognition Dataset and the outperform accuracy reached 99.83%. Khraief et al. proposed a multi-stream fall detection system using an RGB-D sensor which is based on CNN in [69]. Their system combines four modalities such as motion, shape, RGB, and depth information. Here, the motion images are based on the optical flow displacement, amplitude, and orientation of optical flow to capture the velocity and the direction. Transfer learning and data augmentation were used to supplement the insufficient training data. They also incorporated the Adam optimizer and cross-entropy loss function. The model was evaluated on three publicly available datasets, namely the Multiple Cameras Fall (MCF), the UR Fall Detection (URFD) and Fall Detection Dataset (FDD). Abobakr et al. presented an integrable, privacy-preserving fall detection system using an RGB-D sensor in [70]. They used deep hierarchical visual representations and complex temporal dynamics features extracted using Residual ConvNet. They also used recurrent LSTM networks to learn temporal dynamics that can differentiate between fall and non-fall events. The model was trained end-to-end using backpropagated gradients. They evaluated their model on a publicly available URFD fall detection dataset and achieved 98% accuracy. Xu and Zhou proposed a home-health fall detection system for seniors based on biomechanical features in [71]. They used 3D skeleton data and the Center of Mass (COM) of different body segments as biochemical features. They employed an LSTM network for fall detection and calculated Line of Gravity (LOG) and Base of Support (BOS). They obtained 97.41% accuracy for the TST Fall detection database v2. Amrita et al. proposed an effective fall detection system using the YOLOv2 network on depth videos in [72]. They calculated parameters such as the subject’s height to width ratio and fall velocity. Their proposed method incorporated CNN.

Classical ML-based Methods: Mazurek et al. proposed a depth silhouette image-based unobtrusive fall detection method using an infrared depth sensor in [73]. They used Kinematic and Mel-cepstrum features that yield highly correct classification results. Three classification algorithms have been used here. These are: SVM, artificial neural network (ANN), and Naive Bayes (NB) classifiers. Tests were conducted on two datasets, namely the IRMTv1 and TSTv2 dataset and 98.6–100% and 93.9–97.7% accuracies were obtained for the combined features. Another technique of human fall detection using depth videos was proposed by Akagündüz et al. in [74]. Their work was based on a shape sequence descriptor called Silhouette Orientation Volumes (SOV). To characterize and classify each action, they combined SOV with Bag-of-Words and an NB classifier. They also used the k-medoids clustering algorithm for codebook generation. Codebook generation is an important influence in Vector Quantization for lossy image compression. They selected SDU-Fall and Weizmann action datasets for their experiment and achieved an overall accuracy of 89.63%. Aslan et al. proposed a shape-based fall characterization method based on depth videos in [75]. They used Curvature Scale Space (CSS) features and Fisher Vector (FV) encoding. CSS is a method of mapping images from three-dimensional space to a space that represents each point as a curvature with regard to the arc-length. They experimented on the SDUFall dataset [76] and achieved an 88.83% accuracy using an SVM classifier and overall accuracy of 64.67% for 6-class action recognition. Bian et al. presented a fall detection technique based on human key joints which uses an infrared depth camera that can operate even in low light or dark conditions in [77]. They employed a Randomized Decision Tree (RDT) algorithm to extract key joints features of the body. They also used an SVM classifier that uses 3D joint trajectory to detect falls. Despite being a low computational cost model, their method returned better accuracy than several other state-of-the-art methods. However, the proposed approach cannot detect a fall which leads to lying on furniture, since the distance between the body and the floor is too high. Kepski and Kwolek proposed a fall detection technique using a KNN classifier which was focused on low computational cost and a reduction in the false-positive signal in [78]. They used an accelerometer to reduce the processing overhead. It led to an almost 0% error after evaluation with more than 45,000 depth images.

Other Feature Engineering-based Methods: Rougier et al. designed a fall detection technique that accepts the depth video sequence as input in [79]. They proposed an occlusion-based method where they used two features– one is human centroid height–relative to body velocity and the ground. Here, it was mentioned that human fall may not be correctly detected if a fall occurs behind any furniture, so they incorporated centroid velocity features, human centroid height relative to the ground, and body velocity. They also incorporated the V-disparity approach. It is constructed by calculating a horizontal histogram of the disparity stereo image. This model has been tested on simulated falls and normal activities (such as walking, sitting down, crouching down). In another study in [80], Nghiem et al. proposed an approach that detects the human head position based on depth video. Here, the fall detection was achieved according to the speed of the head, the body centroid, and their distance to the ground. They used a modified Histogram of Oriented Gradient (HOG) approach. This approach was evaluated on a dataset of 30 fall, 18 crouch, and 13 sit-down actions. This approach cannot work in cases of occlusion because the algorithm needs to compute the distance to the ground. Zhang et al. presented a viewpoint-independent statistical method for fall detection based on depth video in [81]. The speciality of this system is that changing the camera viewpoint is easy and requires less effort, as there is no need to train for new data. They used a background subtraction algorithm for person detection with features such as distance from the floor, acceleration, and three more additional features (e.g., smallest head height, total head drop, and fraction of frames) for better accuracy. In [82], Kepski and Kwolek focused on a low computational cost fall detection system. They used three main methods which are–Random Sample Consensus (RANSAC) algorithm, v-disparity images, and Hough transform. They also extracted a ground plane to calculate the distance of a person to the ground. Here, the fall alarm will be raised based on the segmented person that uses updated depth-reference images. Gasparrini et al. proposed a depth-based privacy-preserving fall detection system using an ad-hoc segmentation algorithm in [83]. They incorporated features such as head–ground and head–shoulder distance gap and head dimension. At first, the depth-frames were preprocessed and then the segmentation technique was applied. After that, the algorithm classifies the pixels and the system recognizes the human subject and detects if a fall occurs or not. Yang et al. proposed a computationally efficient spatio-temporal context tracking technique using Kinect-based 3D depth images to develop a powerful fall detection system in [84]. In the pre-processing phase, they estimated the parameters of the Single Gauss Model (SGM) and extracted silhouettes. After that, they applied the dense spatio-temporal context (STC) technique to track the head position and the distance from the floor. Their method can also help to detect fall incidents in various orientations. Yang et al. proposed an indoor fall detection method for elderly people using 3D depth images in [85]. They used a median filter to pre-process depth images and then converted the images into a disparity map. A least-square method was used to estimate the floor plane equation. The silhouettes in each depth image were obtained by employing the background frames subtraction technique. To detect the fall, they further calculated centroids of the human body and the angle between the human body and the floor plane. The method is based on threshold detection, which avoids feature extraction and classification. Chen et al. proposed the asymmetry principle to recognize accidental fall and used the OpenPose [86] technique to extract skeleton information of the human body in [87]. Here, falls were identified based on three parameters. These are: a. speed of descent, b. the human body centreline angle with the ground and c. width-to-height ratio of the body. Their method obtained a 97% accuracy rate.

For faster and easier understanding, we have projected the above reviewed work in Table 1.

#### 3.1.2. Fall Detection with Gait Parameter

Here, we reported several works on fall detection which used gait parameters.

DL-based Methods: Murthy et al. proposed a gait-based person fall detection technique using deep CNN in [88]. They used gait energy images (GEI) for the input that preserves the dynamic and static information of a gait sequence. Their model obtained classification results with an accuracy of 99.1% and a prediction ratio of 98.64%. M.Amsaprabhaa et al. developed a Multimodal SpatioTemporal Skeletal Kinematic Gait Feature Fusion (MSTSK-GFF) classifier for fall detection in [89]. They used two sets of spatiotemporal kinematic gait features generated from a SpatioTemporal Graph Convolution Network (STGCN) and 1D-CNN network model. They applied a hyena optimizer to update the network’s weights. The experiments were evaluated using two datasets, namely UR Fall detection (URFD) and a self-build dataset and achieved accuracies of 96.53% and 95.80%, respectively.

Classical ML-based Methods: Xu et al. proposed a method based on skeleton tracking and human body gesture recognition in [90]. They used an optimized BP neural network to realize fall detection. They also used the NITE body tracker for testing and the Kinect V2 sensor to process human joints. Their aim was to recognize activities such as standing, sitting and lying positions. The experiment used the MSRDailyActivity3D dataset and achieved a drop test accuracy of over 98%. Dubois and Charpillet developed a system to prevent falls of seniors by analyzing the displacement of the center-of-mass of the persons in [91]. They extracted three gait parameters to assess fall risk, which are: length and duration of steps and the speed of the gait. They adopted a Hidden Markov Model (HMM) for the activity analysis. Parajuli et al. presented a fall detection system by analyzing gait and posture data, such as data on walking, sitting, standing, etc., in [92]. To analyze these gait and posture data, they used SVM. The Radial Basis Function(RBF) kernel has also been used here. They collected the following four datasets: normal walking, abnormal walking, standing, and sitting for model evaluation. They performed posture recognition (sitting versus standing) and gait recognition (normal walking versus abnormal walking).

Other Feature Engineering-based Methods: Stone and Skubic investigated Fall detection using gait analysis by measuring temporal and spatial gait parameters in [93]. They used a Vicon motion capture system for ground truth. They also used the background subtraction algorithm to extract the foreground. They collected 18 walking sequences from three participants for model testing. Another study conducted by Stone and Skubic using two types of data, i.e., anonymized video data and depth imagery data, can be found in [94]. They computed stride-to-stride gait variability and compared it with the Vicon system. They also used the background subtraction technique to extract silhouettes from the raw images. In total, 18 walking sequences were collected for model evaluation. Baldewijns et al. presented a non-intrusive gait analysis technique by measuring step length and time and validated it using GAITRite in [95]. They further determined the center of mass using the mean position and also used connected component analysis to remove noises. Table 2 shows the above reviewed work in short.

### 3.2. Activity Analysis

Using an activity pattern analysis, we can analyze seniors or patients and can detect any health problems they might be having. An activity analysis can predict heart attacks, falls, and many other diseases. It will be more useful if we analyze it using gait parameters. If a person is experiencing certain health issues, their gait is affected more than any other activities. So, we integrated th gait parameter for a more effective analysis. In the following section, we report some works on activity analysis through depth image classification techniques without and with gait parameters.

#### 3.2.1. Activity Analysis without Gait Parameter

Here, we have reported several important research works on activity pattern analysis without gait parameters.

DL-based Methods: Jaouedi et al. presented the novel approach of a Human Activity Recognition (HAR) system based on Skeleton Features and a DL model in [96]. For activity classification, they used Gated Recurrent Unit (GRU)-based RNN with the Kalman filter to improve its cognitive capability. They also used transfer learning CNN for feature presentation. Their proposed system used three types of features, namely visual, temporal, and 2D human skeleton. They used the HDM05-122 dataset for the evaluation and achieved an accuracy of 91.5%. Phyo et al. proposed a DL-based intelligent HAR system using Motions of skeletal joints in [97]. They used two features. The first one is motion history which was extracted using Color Skeleton Motion History Images (Color Skl-MHI). The second one is the relative distance which was obtained from the Relative Joint Images (RJI). They used deep CNN (3D-DCNN) to recognize human actions. They aimed to develop this as a consumer electronic product by reducing its computational cost. Skeletal joints were used as inputs. They achieved a 97% based on an evaluation with UTKinect Action-3D and CAD-60 datasets that include daily activities such as drinking water, answering the phone, and cooking. Bagate and Shah proposed an RGB-D sensor-based HAR system using CNN in [98]. Two features were used here. One is a spatial feature (skeletal joints) and the other one is temporal features (i.e., sequential frame). Their model reduces the number of convolution layers and provides better results compared to other LSTM-based models. Their work focused on body gestures, motion, and the identification of multiple activities performed at the same time. They used the SBU kinect interaction dataset and considered a confusion matrix for evaluation and achieved 85% accuracy. GU et al. presented a depth MHI (motion history images)-based DL-model for the HAR system in [99]. They used depth sequences as the input and a confusion matrix for model evaluation. ResNet-101 was chosen as the DL model. The proposed model was evaluated using both RGBD-HuDaAct and NTU RGB+D datasets and achieved a top-1 accuracy of 84.44% and 67.97% for each dataset.

Uddin et al. proposed a facial expression recognition system (FER) to develop a care system for seniors using depth video data in [100]. They used a local directional position pattern (LDPP) to extract the local directional strengths feature for each pixel. They also incorporated a principal component analysis (PCA) and generalized discriminant analysis (GDA) to improve the feature extraction process. They considered the following six facial expressions: anger, happy, sad, surprise, disgust, and neutral. Finally, they used a Deep belief network (DBN) for recognition and achieved an accuracy of 96.67%. X. Ji et al. proposed a novel and efficient method for human action recognition using depth map sequence and 3D ResNet-based CNNs in [101]. To capture the appearance and motion, they developed a depth-oriented gradient vector (DOGV) for short-term and CNNs-based backbone for longer periods. The experimental results proved that the proposed approach can achieve state-of-the-art performance on four benchmark datasets (NTU RGB+D, NTU RGB+D 120, PKU-MMD and UOW LSC). To evaluate the proposed method, they employed random cross subjects and random cross sample protocols. S.K.Yadav et al. proposed an activity recognition and fall detection system using a deep convolutional long short-term memory (ConvLSTM) network in [102], which involves a sequential fusion of convolutional neural networks (CNNs), long short-term memory (LSTM) networks, and fully connected layers. They used geometrical and kinematic features to construct the novel guided features. Only skeleton joints coordinates along with suitable features were used for inputs in the model. They also used cross-entropy and softmax activation to obtain the model loss and performance measures. This proposed model has been evaluated on the KinectHAR video dataset and achieved an accuracy of 98.89%.

Classical ML-based Methods: Jalal et al. presented a depth video-based HAR framework in [103] using multi-features and embedded HMM. It has the ability to track human body parts in real-time. Here, the temporal motion identification method was used to track human movements. They made their own dataset for evaluation. Kamal et al. proposed a depth video-based robust method using spatio-temporal features and modified the hidden Markov model (M-HMM) in [104]. For classification, they fused the depth shape and temporal joints features. They also used depth silhouettes and body joint information. Silhouettes were extracted from noisy background subtraction and floor removal techniques. They evaluated their model using two datasets, namely MSRDailyActivity3D and IMDailyDepthActivity and achieved accuracies of 91.3% and 68.3% for each dataset. Farooq et al. proposed an RGB-D Map-Based Human Tracking and Activity Recognition system using the K-means clustering algorithm in [105]. They extracted depth silhouettes and body skin joints features. The human joint point was computed using the Distance Position and Centroid Distance Features. They evaluated the model using their own recorded depth silhouette datasets and achieved 89.72% accuracy. The dataset contains nine activities, such as walking, sitting down, exercise, preparing food, standing up, cleaning, watching TV, eating a meal and lying down.

Chen et al. presented an action recognition method based on depth motion maps(DMMs) in [106]. They employed local binary patterns (LBPs) as well as a kernel-based extreme learning machine (KELM) for their model. Their model was tested with two different datasets, namely the MSRAction3D and MSRGesture3D datasets.

Jalal et al. designed a lifelogging HAR (Human Activity Recognition) system for seniors in [107]. They captured depth silhouettes that produce human skeletons with joint information. They first collected data using a depth camera; then, features were generated. Finally, they used the HMM for training and then began recognition to produce life logs. Life logs contain records of daily human activity (e.g., activity name, time, number of occurrences, etc.) using a video camera. They evaluated their system using life-logging features against the principal and independent component and achieved satisfactory results compared to the conventional approaches. They also conducted their experiment on the MSRDailyActivity3D dataset [108] and achieved a promising result.

Jalal and Kamal presented a depth-based human activity recognition model using life logs in [109]. They used HMM as an activity recognizer. They also computed a set of magnitude and direction angle features to compute body points. The experimental results show an improvement in the accuracy rate (i.e., 89.33%) over other conventional systems. Kospmopoulos et al. investigated human behavior based on depth and color videos using a fused time series classifier in [110]. They extracted forward and backward feature vectors from depth videos and color videos. They also extracted human blob features from color videos and used these combined features as inputs for the classifier. They incorporated the HMM in their proposed system. They tested their model on the RGBD-HuDaAct dataset which includes twelve activities, including sit down and stand up. M.F. Bulbul and H. Ali proposed a depth video oriented towards human action recognition approach using the KELM classifier in [111]. They obtained motion history images (MHIs), static history images (MHIs) and a 2D auto-correlation gradient feature vector. They also used the LBP algorithm to represent motionless images as binary-coded images. This approach was assessed on MSRAction3D, DHA, and UTD-MHAD datasets and achieved accuracies of 97.44%, 99.13% and 88.37%. The depth images of each dataset were used directly in the model without any segmentation.

Others Feature Engineering-based Methods: Srivastav et al. proposed an end-to-end solution incorporating a super resolution image estimator and a 2D multi-person pose estimator in a joint architecture for Human Pose Estimation (HPE) problem on depth images in [112]. Their architecture is a modification of the RTPose network [113]. They used the MVOR dataset for evaluation and achieved an improved accuracy, of 6.5% above the baseline RTPose 64 × 48 and 3.6% better than RTPose 80 × 60.

Above reviews have been shown in Table 3 shortly.

#### 3.2.2. Activity Analysis with Gait Parameter

Here, different activity pattern analysis techniques using gait parameter have been explored.

In [114], Uddin and Kim proposed a DL-based human gait posture recognition system based on depth video using Local Directional Patterns (LDP) for feature extraction. After that, a DBN was trained to recognize postures. The pre-training was performed based on Restricted Boltzmann Machine (RBM) and then weights were applied with the fine-tuned algorithm. They built a depth gait database for normal and abnormal gait activities that consists of 1000 images.

Bari and Gavrilova proposed a DL-based gait recognition model in [115]. They introduced two new features, namely the Joint Relative Triangle Area (JRTA) and Joint Relative Cosine Dissimilarity (JRCD). These are the view and pose invariant geometric features. To enhance the performance of the system, they incorporated the Adam optimizer. They used two publicly available benchmark datasets, namely the UPCV gait dataset and Kinect gait biometry dataset and achieved accuracies of 95.30% and 98.08%. Wang et al. proposed a multichannel CNN-based human gait recognition scheme in [116], where they introduced a new feature called TriTuple Gait Silhouettes(TTGS). They achieved multichannel abilities by incorporating more input channels. The evaluation was performed with two gait datasets, namely CASIA and OU-ISIR. Uddin et al. presented a depth image-based human activity recognition system using HMM in [117]. This system analyzes daily activities and generates an alarm if it detects abnormal gait. They applied PCA and ICA (Independent Component Analysis) to extract spatiotemporal features. The proposed system achieved an average accuracy of 92.50% for normal and 95% for the abnormal gait recognition.

Gabel et al. presented a low-cost, non-intrusive gait analysis system based on a Kinect sensor and software development kit (SDK) in [66]. They measured arm kinematics and used the whole body to measure stride intervals. Supervised learning was used to measure gait parameters. Skeleton information was converted into a large set of features, which are fed into a regression tree to predict the values of interest. To learn the regression model, they used the Multiple Additive Regression Trees (MART) algorithm.

In another study in [118], Nandy and Chakraborty proposed a new approach of human gait analysis to find an intrinsic gait posture using the Kinect Xbox device. They used an NB classifier for classification and minimized segmentation errors using the automated background subtraction technique. The proposed system has been compared with the Intelligent Gait Oscillation Detector (IGOD) [119] and produced encouraging results. Chaaraoui et al. proposed an abnormal gait analysis method using the Bag of KeyPoses classification algorithm that relies on skeletal pose representation in [120]. They used the novel spatio-temporal feature to locate skeletal joints and the motion’s age. Their approach mainly focused on gait monitoring, rehabilitation and the early diagnosis of cognitive impairment. After evaluation on a publicly available dataset from the SPHERE project [121], they were able to detect abnormal gait with high performance.

Another Kinect-based gait analysis with a visualization system was presented in [122] by Dao et al. that captured the human skeleton and generated a Bio-vision Hierarchy (BVH) file. Their system contains the following two components: motion analysis and visualization. The motion analysis component processes and encodes data into the BVH file and assesses the extracted gait feature. The motion visualization component helps to visualize the walking motion. Their proposed model used a linear SVM classifier for the gait classification. They used their own dataset that consists of 20 normal and 30 abnormal walking motions and achieved 88% accuracy, which is higher than the existing performance accuracy rate (86.63%).

Another privacy-preserving low-cost system was proposed by Dubois and Charpillety in [123] which analyzes the displacement of seniors by applying local computing. They measured gait by analyzing the trajectory of the centre of mass of the person and used the HMM for fall detection. Their proposed system extracted features such as the centre of mass and vertical distribution silhouette. Bei et al. introduced a new concept called ‘Gait symmetry’ to measure the similarity of leg swing motion in [124]. They extracted spatio-temporal parameters, such as the step length and gait cycle using a zero-cross detection method. They also extracted leg swing characteristics formed by hip, knee, and ankle joints. They applied the K-means and Bayesian method in their model. They mainly focused on gait analysis using frontal walking sequences and mostly extracted very simple features, e.g., the step length and gait cycle. They also applied gait symmetry to achieve better accuracy.

Table 4 shows the above reviews in short.

## 4. Survey of Benchmark Datasets

Data are the fuel for any data-driven computing engine such as DL-based computing. To develop a useful in-home care system for seniors, the predictive model part of the system needs to be trained using a dataset that is preferably labeled. Therefore, a survey is necessary to explore the availability of different high-quality datasets. Below, we review some benchmark datasets.

Cheng et al. proposed the first multi-view RGBD dataset, ACT4${}^{2}$ in [125], for human daily action analysis. It contains 6844 actions clips from four viewpoints and two sources. The aim of the dataset was to facilitate smart houses or e-healthcare by focusing on the different daily activities of humans. They invited 24 people to perform 14 different activities such as sit-down, sit-up, drink, etc., in order to create the dataset. Another dataset, namely Kinect 3D Active (K3Da) for human motion analysis was released by Leightley et al. in [126] using Kinect One. It collects data from different ages of people ranging from 18 to 81 years. A total of 54 participants were chosen to perform different types of tests, including walking, sitting, standing, and other balance assessments. Shahroudy et al. introduced the NTU RGB+D dataset for human action recognition using Microsoft Kinect sensor in [127]. It consists of 56,880 RGB-D video items captured from 40 different human subjects, with their ages ranging from 10 to 35 years. The dataset has 60 different classes including eating, falling, hugging, etc. Liu et al. presented a skeleton-based human action understanding dataset PKU-MMD in [128]. It contains color and depth images, infrared sequences, and skeleton joints. The dataset contains 1076 long video sequences performed by 66 subjects ranging between 18 and 40 years old. It also contains around 20,000 action instances, 5.4 million frames, and 3000 min of videos. It has 51 action classes, such as drinking, hugging, waving hands, shaking hands, etc. Aloba et al. developed a child and adult Motion Capturing dataset named Kinder-Gator using Kinect V1.0 that tracks joints such as the elbows, knees, hips, etc. in [129]. They collected 58 different motions such as hand waving, kicking a ball, etc., performed by 10 children (ages 5 to 9) and 10 adults (ages 19 to 32). This dataset also includes RGB videos and 1159 motion trials. Jang et al. released a dataset called ETRI-Activity3D to recognize daily seniors’ activity using the Kinect v2 sensor in [130]. It contains 112,620 samples of 100 people performing 55 daily activities. Out of 100, the age of 50 people is in the range of 64 to 88 years and others were in their 20s. They used various subjects of different age ranges to properly observe and understand the behavior of individuals. The dataset includes RGB videos, depth maps, and the skeleton sequences of 25 body joints. Fiorini et al. proposed a gesture and activity recognition dataset named VISTA, which is a combination of inertial sensor and depth camera data in [131]. The dataset includes 7682 action instances for the training phase and 3361 action instances for the testing phase. The dataset includes basic gestures, such as walk, ADL, drink, eat, brush teeth, use laptop etc., and scenes such as having lunch, house cleaning, relaxing, etc. Table 5 shows the above reviewed datasets in short.

## 5. Discussions and Future Scopes

In-home monitoring systems for seniors have become necessary requirements. Several methods have been proposed over the years. As shown in Section 3, frequently used techniques are DL, HMM, SVM, and NB classifiers, etc. Different types of features such as human joint information, center-of-mass, silhouettes, spatio-temporal, various distance, etc., are extracted using different techniques. The datasets that are frequently used are MSR DailyActivity3D, SDUFall dataset, etc.

All the selected works which are discussed in Section 3 are summarized in Table 1, Table 2, Table 3 and Table 4. In these four tables key points, features, and used computing techniques are mainly mentioned. The data in these tables have been arranged year-wise in descending order so that the latest works can easily be found.

Additionally, we compare different methods based on the accuracy, condition and activities which are shown in Table 6. We also describe the datasets in terms of accuracy and the drawbacks of the methods in Table 6.

**Figure 4:**
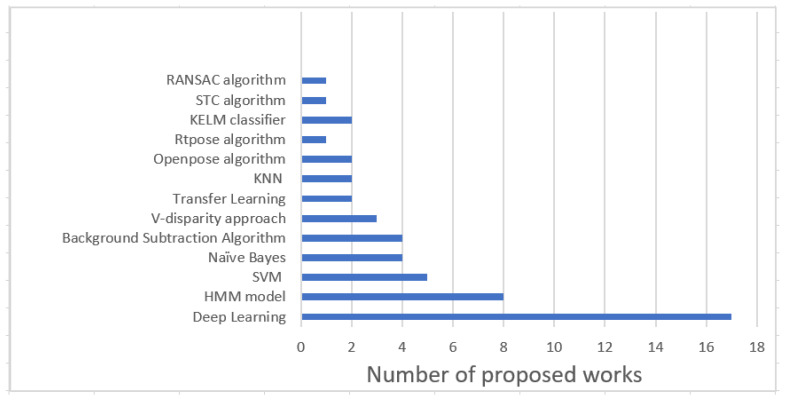
Different Computing Techniques used from the last decade.

It can be observed from Figure 4 and the tables provided above that ML-based techniques are frequently used. These ML techniques are SVM, NB, HMM, and DL.

Although recently proposed techniques are mostly DL-based, the number is not exceptionally high. If we observe the bars in the graph mentioned in Figure 4, we can observe that the data-driven approaches are gaining popularity. These observations are based on the articles published by major publishers in the last decade.

Many researchers have proposed in-home monitoring techniques with depth sensors, but these methods still present various challenges which need to be overcome. Moreover, researchers rarely use local and federated computing methods. These methods might be useful to overcome several challenges regarding latency, privacy, and data security. As in the healthcare sector, the dataset might not be suitable or available for end-to-end training; transfer learning can then be used to train the model with fewer data. Transfer learning is an ML method where a model is trained to perform task A; then, the trained model is deployed to perform a similar task, labelled Task B. For the second task, the previous model acts as a good starting point. In short, the pre-trained model is reused to solve a new related problem. Moreover, transfer learning is suited to DL as deep transfer learning (DTL) is a highly effective data-driven approach.

There also exist recently proposed meta-learning techniques such as Few Shot Learning (FSL). FSL works with less labeled data. This is useful when training instances are either rare or costly. A typical example is drug discovery, i.e., discovering various properties of new molecules to develop a new useful drug. Another example is in the medical field where a small number of X-ray images of a particular part of the body are available. In these fields, collecting a lot of data to train a neural network is very difficult. FSL could be used in these situations. There are many application where FSL has been used successfully, such as in face verification, character recognition, video classification, motion prediction, etc., [150].

IoTs capability could be enhanced by deploying a pre-trained model. It can now process some of the data in the edge and can reduce the workload in the cloud. Another problem often faced by these systems is a disturbance in network connectivity. So, a backup system is required to perform recovery. The algorithms could also be optimized to produce faster outputs. The privacy issue of the in-home care system could be resolved using depth sensors; however, the data security issue has yet to be resolved.

A large storage system is needed to store all the data which are generated due to continuous monitoring. These huge amounts of data can be utilized for the long-term health assessment of seniors. Imbalance and biases in the dataset are concerns which may be handled via different techniques. Some qualitative data also needed to be analyzed to understand whether our seniors are comfortable or not with this continuous technology-based monitoring system. The monitoring system could be modified according to the feedback provided by the seniors to make it more friendly. Affordability is the most crucial feature of any in-home care system. It should be designed in such a way that it becomes affordable without sacrificing the quality of life-saving features of the system. In the future, the research direction could help to find solutions for the above-mentioned problems.

## 6. Conclusions

In this paper, we reviewed different computational techniques which were proposed to develop in-home monitoring systems for older adults which primarily use depth sensor data. At first, we reviewed fall detection with and without the gait-based depth image classification technique; we then reviewed the activity pattern analysis using the same classification. Although existing in-home senior monitoring systems provide various useful features as well as high levels of accuracy in predicting various events, some basic challenges (e.g., privacy, security, latency, storage, etc.) are yet to be overcome. Ideal in-home care for older adults should facilitate them in their homes with minimum cost, ensuring their privacy as well as assisting them in an emergency situation. We strongly believe that the newly proposed techniques such as transfer learning, few-shot learning, incremental learning, etc., should be incorporated into such a system for faster processing and accurate activity detection with a low computational burden. Edge computing and federated learning may mitigate the challenges that cloud computing has. However, we may need to use the cloud server, as without this, the process cannot be completed for many reasons. IoT devices and sensors need to be more intelligent so that they can achieve faster processing and remove the overhead of the edge as well as the cloud server. This paper shows the methods and approach that researchers used in the last decade. Furthermore, we provided the most recent work first in the tables to make it easier to review the latest progress. The evaluation of the use of ML, DL and other feature engineering methods is presented in the graph. Overall, this paper provides a review of the current techniques, future scopes, challenges and some solutions for in-home care systems for seniors using depth sensor imagery.

## Figures and Tables

**Table 1:** Overview of the fall detection using depth imagery without gait parameter from the last decade.

Study with Year	Key Points & Features	Computing Technique Used
Amrita et al. [72], 2022	used subject’s height to width ratio and fall velocity.	CNN
Chen et al. [68], 2022	Used 2D and 3D poses from depth video sequences.	CNN
Z.Chen et al. [87], 2020	Used symmetry principle and calculated speed, angles and width-to-height ratio.	OpenPose algorithm
Khraief et al. [69], 2020	Combines motion, shape, color and depth information. Used transfer learning and data augmentation technique to deal with training data.	CNN and Transfer learning
Abobakr et al. [70], 2018	Deep hierarchical visual representation and complex temporal dynamics using residual ConvNet.	Recurrent LSTM
T. Xu and Y. Zhou [71], 2018	Accelerated velocity of left-of-Mass (COM) and 3D skeleton data	LSTM network
Mazurek et al. [73], 2018	Kinematic feature and mel-frequency-cepstrum-related features	SVM, ANN and Naïve Bayes classifier(NBC)
Akagunduz et al. [74], 2016	Silhouette Orientation Volume (SOV) feature, bag-of-words approach for characterization, K-medoids clustering for constructing codebook.	Naïve Bayes classifier
Yang et al. [85], 2016	Floor plane and shape information, as well as the threshold were calculated. Depth images were preprocessed by median filter.	V-disparity map and least square method
Aslan et al. [75], 2015	Curvature Scale Space (CSS) features and Fisher Vector (FV) encoding	SVM classifier
Yang et al. [84], 2015	Extracted silhouette with SGM and calculates threshold for the distances from head and centroid to the floor-plane.	STC algorithm
Gasparrini et al. [83], 2014	Uses head–ground and head–shoulder distance gap and head dimension features and calculates threshold for fall.	Ad-Hoc segmentation algorithm
Bian et al. [77], 2014	3D human body joints extraction and tracking using RDT algorithm.	SVM classifier
M. Kepski & B. Kwolek [78], 2014	Accelerometer and features such as head–floor distance, person area and shape’s major length to width were used.	KNN classifier
M. Kepski & B. Kwolek [82], 2013	Extracts ground plane distance and uses segmented depth reference images.	v-disparity, Hough transform and the RANSAC algorithm
Zhang et al. [81], 2012	Combines viewpoint invariance, simple system setup, and statistical decision making. Uses features such as distance from the floor and acceleration and computed threshold.	Background Subtraction algorithm
Nghiem et al. [80], 2012	Uses centroid speed and position as the main features, and incorporates the head detection algorithm	Modified HOG Algorithm
Rougier et al. [79], 2011	Uses features such as human centroid height relative to the ground and body velocity. Ground plane detection and segmentation was were performed.	V-disparity approach

**Table 2:** Overview of fall detection using depth imagery with gait parameter from the last decade.

Study with Year	Key Points & Features	Computing Technique Used
M.Amsaprabhaa et al. [89], 2022	used spatiotemporal kinemetic gait features.	CNN.
Murthy et al. [88], 2021	Uses gait energy images	Deep convolutional neural network (DCNN)
Xu et al. [90], 2019	Skeleton tracking technology of Microsoft Kinect v2 sensor, Body tracker (NITE)	Optimized BP neural network
Baldewijns et al. [95], 2014	Calculates step length and time, centre of mass (COM), mean position, etc. Used connected component analysis to remove noisy pixels	Player detection algorithm
A. Dubois & F. Charpillet [91], 2014	Extracted length and duration of steps and speed of the gait, tracks centre-of-mass.	Hidden Markov Model (HMM)
Parajuli et al. [92], 2012	Measures gait and change in posture from sitting to standing or vice versa. Data transformation, cleaning and reduction were performed.	SVM classifier
E.E. Stone & M. Skubic [94], 2011	Measures stride-to-stride gait variability and assesses the ability of the two vision-based monitoring systems.	Background subtraction technique
E.E. Stone & M. Skubic [93], 2011	Measures temporal and spatial gait parameters, also measures walking speed, stride length, stride time, etc.	Background subtraction algorithm

**Table 3:** Overview of the activity analysis using depth image classification without the gait parameter from the last decade.

Study with Year	Key Points & Features	Computing Technique Used
S.K. Yadav et al. [102], 2022	Used geometrical and kinematic features.	CNN, LSTM, Fully connected layer.
X. Ji et al. [101], 2021	Used frame-level feature termed depth-oriented gradient vector(DOGV) and captured human appearance and motion.	3D ResNet-based CNN.
M.F. Bulbul and H. Ali [111], 2021	Motion and static history images were used. LBP algorithm and GLAC descriptor were also used.	KELM classifier.
Jaouedi et al. [96], 2020	Uses visual, temporal and 2D human skeleton features and kalman filter. A hybrid combination of different models was used.	RNN, CNN, Transfer learning.
Srivastav et al. [112], 2019	Integration of a super-resolution image estimator and a 2D multi-person pose estimator in a joint architecture	Modified RTPose network
Phyo et al. [97], 2019	Motion history images extracted using Color Skl-MHI and relative distance using RJI. Used image processing.	DCNN
A. Bagate & M. Shah [98], 2019	Uses spatial, i.e., skeletal joints and temporal features and reduces the convolution layer.	Convolution Neural Network
Gu et al. [99], 2018	MHI and evaluated on both 3D human action datasets RGBD-HuDaAct and NTU RGB+D.	ResNet-101
Uddin et al. [100], 2017	Local directional strengths features were extracted by PCA, GDA and LDPP	Deep Belief network (DBN)
Jalal et al. [103], 2017	Extracts 3D human silhouettes and spatiotemporal joints and several other features are also fused to make some changes.	Hidden Markov Model (HMM)
Chen et al. [106], 2015	Depth motion maps (DMMs) and local binary patterns (LBPs) were used to capture motion cues and to achieve compact feature representation.	KELM classifier
Jalal et al. [107], 2014	Skeletal model and joint position were collected and life logs that contains human daily activities were generated.	Hidden Markov Model (HMM)
Jalal et al. [109], 2014	Human skeletal images with joint information were produced that generate life logs and also utilize magnitude and directional angular features from the joint points.	Hidden Markov Model (HMM)
A. Jalal & S. Kamal [110], 2013	Fused color and depth video, extracted forward and backward feature vectors and calculated some other features that describes human body information.	Hidden Markov Model(HMM) and Fused time-series classifier
Kamal et al. [104], 2016	Spatial depth shape and temporal joints features were fused. Human silhouettes extracted using noisy background subtraction and floor removal techniques.	Modified Hidden Markov model (M-HMM)
Farooq et al. [105], 2015	Extracts depth silhouettes & body skin joint features using distance position and centroid distance.	K-means clustering

**Table 4:** Overview of activity analysis using depth image classification with gait parameter.

Study with Year	Key Points & Features	Computing Technique Used
Wang et al. [116], 2020	Trituple gait silhouettes(TTGS) feature	Multichannel CNN
A.H. Bari & M.L. Gavrilova [115], 2019	Two features of joint relative triangle area (JRTA) and joint relative cosine dissimilarity (JRCD)	DL model
Bei et al. [124], 2018	Step length and gait cycle extracted using the zero-crossing detection method, combining gait symmetry and spatiotemporal parameters.	K-means and Bayesian method
A. Dubois & M. Charpillet [123], 2017	Centre of mass and vertical distribution silhouette features were extracted, measuring the degree of frailty.	Hidden Markov model (HMM)
M.Z. Uddin & M.R. Kim [114], 2016	Local directional feature and Restricted Boltzman Machine (RBM)	Deep Belief Network (DBN)
Dao et al. [122], 2015	Generates BVH file, uses motion analysis, motion visualization and integrates data capturing, data filtering, body reconstruction, and animation.	SVM classifier
Chaaraoui et al. [120], 2015	Joint motion history feature (JMH) encodes spatial and temporal information.	BagOfKeyPoses algorithm
A. Nandy & P. Chakraborty [118], 2015	Knee and hip angular movement, using IGOD biometric suit. Features were measured by Fisher’s discriminant analysis.	Naïve Bayes’ rule and k-Nearest Neighbor
M. Gabel et al. [66], 2012	Measures arm kinematics, stride duration, used 3D virtual skeleton to extract body gaits	Supervised learning approach, MART algorithm, and regression trees
Uddin et al. [117], 2011	Spatiotemporal features were extracted and feature space was generated using ICA and PCA, with the background removed by Gaussian probability distribution function.	Hidden Markov Model (HMM)

**Table 5:** Overview of the in-home health dataset using depth sensor from the last decade.

Dataset	Year	Activity	Brief Description	Recently Used in
VISTA dataset [131]	2022	Basic gestures and daily activities	Contains 7682 action instances for the training phase and 3361 action instances for the testing phase.	New dataset (no published work available)
ETRI-Activity3D [130]	2020	Daily seniors’ Activity	It contains 112,620 samples including RGB videos, depth maps, and skeleton sequences and 100 subjects performed 55 daily activities.	[132,133,134]
Kinder-Gator [129]	2018	Human Motion Recognition	The dataset contains joint positions for 58 motions, such as wave, walk, kick, etc., from ten children (ages 5 to 9) and ten adults (ages 19 to 32). It also contains 19 RGB videos and 1159 motion trials.	[135,136,137]
PKU-MMD [128]	2017	Human Action Analysis	Collection of 1076 long action sequences and 51 action classes. It also contains around 20,000 action instances and 5.4 millions frames.	[138,139,140]
NTU RGB+D [127]	2016	Human Activity Analysis	Consists of 60 different classes and 56,880 video samples captured from 40 distinct human subjects using 80 camera viewpoints.	[141,142,143]
K3Da [126]	2015	Human Motion Analysis	It includes motions collected from fifty-four participants of young and older men and women aged from 18–81 years. It captured balancing, walking, sitting, and standing.	[144,145,146]
ACT4${}^{2}$ [125]	2012	Human Daily Action	The dataset contains 6844 action clips with both color and depth information, collected from 4 viewpoints.	[147,148,149]

**Table 6:** Comparison of some Methods with activities and their drawbacks.

Study with Year	Methods	Dataset with Accuracy	Used Resources	Running Time	Activities	Conditions	Drawbacks
In [102], 2022	ConvLSTM	KinectHAR (98.89%)	NVidia TITAN-XGPU.	Not mentioned	Standing, walking slow, walking fast, sitting, bending, fall, and lying down activities.	Independent of the pose, position of the camera, individuals, clothing, etc.	Provides very high accuracy but is costly due to its complex model structure.
In [111], 2021	KELM Classifier	MSRAction3D (97.44%), DHA (99.13%) and UTD-MHAD (88.37%)	Desktop with intel i5-7500 Quad-core processor and 16 GB RAM	731.4 ± 48.8 ms/40 frames	Sport actions, daily activities, and training exercises.	In consistent real-time operation, it processes 40 depth images in less than a second.	This method did not remove the noise to improve the performance, thus, some misclassifications were observed in activities such as waving, clapping, skipping, etc.
In [150], 2020	Multichannel CNN (MCNN)	CASIA gait B and OU-ISIR	Not mentioned	Not mentioned	Dynamic gait recognition	When there is a pause in the walking cycle, the leg is agile, walking wearing coats and walking carrying bags.	Performance reduces as they used only silhouette images, though they obtained original gait videos.
In [97], 2019	Image Processing and Deep Learning	UTKinect (97%) and CAD-60 (96.15%)	Not mentioned	0.0081 s (UTKinect Action-3D)	Daily activities such as drinking water, answering the phone, and cooking.	In real time embedded systems	Complex actions related to health-problems, such as headaches and vomiting cannot be detected with this approach.
In [124], 2018	K-means and Bayesian	Own dataset of 120 walking	Lenovo Y700-15ISK with an i7-6700HQ CPU and 16G RAM	Not mentioned	Kinematic leg swing characteristics in combination with spatiotemporal parameters such as the step length and gait cycle.	Focused on gait analysis using frontal walking sequences.	Variation of clothing of the object decreases the accuracy.
In [105], 2015	K-means Clustering	Own dataset with 9 different activities. (89.72%)	PC as Intel Pentium IV 2.63GHz having 2GB RAM	Not mentioned	Walking, sit down, exercise, prepare food, stand up, cleaning, watching TV, eating meal and lying down.	In complex activities such as self-occlusion, overlapping among people, and hidden body parts, etc.	Comparatively low accuracy rate as it handles complex activities.

## Data Availability

Not applicable.
